# Next-generation sequencing-based mRNA and microRNA expression profiling analysis revealed pathways involved in the rapid growth of developing culms in Moso bamboo

**DOI:** 10.1186/1471-2229-13-119

**Published:** 2013-08-21

**Authors:** Cai-yun He, Kai Cui, Jian-guo Zhang, Ai-guo Duan, Yan-fei Zeng

**Affiliations:** 1 State key laboratory of tree genetics and breeding, Research Institute of Forestry, Chinese Academy of Forestry, Beijing 100091, China; 2 Key Laboratory of Tree Breeding and Cultivation, State Forestry Administration, Research Institute of Forestry, Chinese Academy of Forestry, Beijing 100091, China; 3 Research Institute of Resources Insects, Chinese Academy of Forestry, Kunming 650224, China

## Abstract

**Background:**

As one of the fastest-growing lignocellulose-abundant plants on Earth, bamboos can reach their final height quickly due to the expansion of individual internodes already present in the buds; however, the molecular processes underlying this phenomenon remain unclear. Moso bamboo (*Phyllostachys heterocycla* cv. Pubescens) internodes from four different developmental stages and three different internodes within the same stage were used in our study to investigate the molecular processes at the transcriptome and post-transcriptome level.

**Results:**

Our anatomical observations indicated the development of culms was dominated by cell division in the initial stages and by cell elongation in the middle and late stages. The four major endogenous hormones appeared to actively promote culm development. Using next-generation sequencing-based RNA-Seq, mRNA and microRNA expression profiling technology, we produced a transcriptome and post-transcriptome in possession of a large fraction of annotated Moso bamboo genes, and provided a molecular basis underlying the phenomenon of sequentially elongated internodes from the base to the top. Several key pathways such as environmental adaptation, signal transduction, translation, transport and many metabolisms were identified as involved in the rapid elongation of bamboo culms.

**Conclusions:**

This is the first report on the temporal and spatial transcriptome and gene expression and microRNA profiling in a developing bamboo culms. In addition to gaining more insight into the unique growth characteristics of bamboo, we provide a good case study to analyze gene, microRNA expression and profiling of non-model plant species using high-throughput short-read sequencing. Also, we demonstrate that the integrated analysis of our multi-omics data, including transcriptome, post-transcriptome, proteome, yield more complete representations and additional biological insights, especially the complex dynamic processes occurring in Moso bamboo culms.

## Background

Biofuels have been proposed as alternatives to relieve the problem of greenhouse gas emissions and energy shortages [1,2]. The increasing demand for lignocellulosic biomass for the production of biofuels make it necessary to exploit fast-growing and high-yield wood resources. Thus, it is of great importance to understand the underlying mechanism of growth especially in height, which is highly correlated with biomass yield, for lignocellulose-abundant plants. As one of the fastest growing lignocellulose-abundant plants on Earth, bamboos can reach their final height of 5–20 m in a single growing season of 2–4 months due to the expansion of individual internodes already present in the buds [3]. To better understand the growth characteristics and physical properties of these bamboos, numerous studies have focused on the general mode of growth, anatomical structure, hormone distributions and chemical and physical characteristics of the culms [4-13], and have observed sequentially elongated internodes from the base to the top [14]. Some internode elongation-associated genes such as *ACO1*, *CENL1*, *EUI1*, *OsGLU1*, *SNORKEL* and *SSD1* have been identified in other plants of Gramineae [15-20], whereas limited molecular information has been provided in the subfamily of bamboos. Despite the sequencing of a set of cDNA [21-26], Expressed Sequence Tags (EST) [27,28], protein expression profiles [29], draft genome [30] , RNA-seq [31] and monoclonal antibody banks [32] in bamboo, the molecular processes underlying rapid internode elongation remain unclear.

Measurements of mRNA and miRNA expression levels, clarity of the regulatory relationships between miRNAs and their corresponding mRNA targets are critical to understanding many pathways and biological systems. With the advent of second-generation sequencing-based technologies such as RNA-Seq and Digital Gene Expression (DGE), it is possible to measure a genome-wide dynamic range of expression in an unbiased manner. These technologies have a high sensitivity and reproducibility compared with existing technologies (e.g. DNA microarrays, cDNA or EST sequencing) [33,34], and will undoubtedly lead to novel insights into plant development and biotic and abiotic stress responses.

To comprehensively understand the molecular processes underlying the rapidly internode elongation of *Phyllostachys heterocycla* cv. Pubescens at the whole genome level, we have studied the differently expressed proteins in different development stages using two-dimensional gel electrophoresis (2D-PAGE) technology [29], here, we performed RNA-Seq, global mRNA and miRNA expression profiling on four different developmental stages and three different internodes. In addition to gaining more insight into the unique growth characteristics of bamboo, we provide a good case study to analyze gene expression of non-model plant species using high-throughput short-read sequencing. And also, the correlation and concordance of transcript and post-transcriptional levels, protein and transcript levels will be studied to explain complex dynamic development processes in the rapid growth of developing culms in Moso bamboo.

## Results

### Anatomical and endogenous hormone variation at different developmental stages of internodes

**Figure 1:**
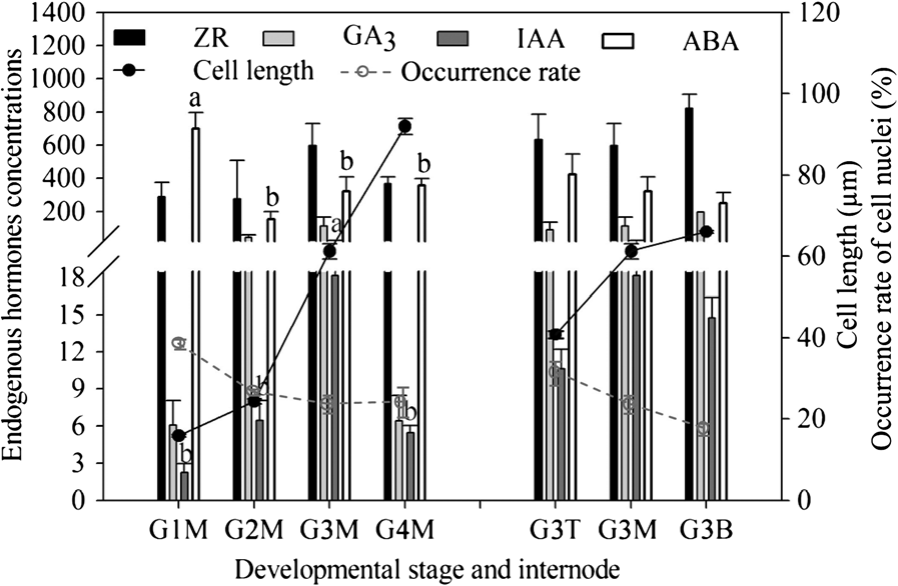
**Variations in cell length (μm), occurrence rate of cell nuclei (%), four endogenous hormones concentrations for *P. heterocycla *culm at different developmental stages and internodes.** Abscisic acid (ABA: ng/g), indole acetic acid (IAA: μg/g), gibberellic acid (GA_3_: ng/g) and zeatin riboside (ZR: ng/g). Four developmental stages or three developmental internodes were defined according to the different unearthed heights of individual plants (0.05, 1.00, 6.00 or 12.00 m; nominated as G1M–G4M, respectively) or different portions (basal internode, G3B; middle internode, G3M; and top internode, G3T) of the same culm (at the G3M stage), respectively. Each data point represents the mean of three biological replicates and three experimental replicates. Different letters on a column with the same pattern indicate significant differences at *P* ≤ 0.05 according to the LSD test. Bars represent SE (n > 150 in cell length and occurrence rate of cell nuclei; n = 9 in four endogenous hormones concentrations).

To better understand the anatomical structure of the culms, transverse and longitudinal sections of culms from the defined development stages were observed under a microscope. Four developmental stages or three developmental internodes were defined according to the different unearthed heights of individual plants (0.05, 1.00, 6.00 or 12.00 m; nominated as G1M–G4M, respectively) or different portions (basal internode, G3B; middle internode, G3M; and top internode, G3T) of the same culm (at the G3M stage), respectively. The number of nuclei declined with culm development (from G1M to G4M stages), and almost disappeared in the later stages (G4M stage). Meanwhile, the length of parenchyma and fiber cells increased continuously with advancing development. Comparison of transverse and longitudinal sections of three developing internodes (G3T, G3M and G3B internodes) in the same developmental stage (at the G3M stage) indicated that the number of nuclei in the tissue increased continuously from basal to apical internodes, while cell length showed the opposite trend (Figure 1). Internode elongation is usually correlated with cell division and elongation. The gradually elongated cell length and gradually decreasing numbers of cell nuclei showed that cell division and elongation occurred simultaneously and thus affecting internode elongation: the former was predominant at initial stages, with the latter predominant at late stages. Thus, the culm showed sequential development from basal to apical internodes throughout the growth period.

The dynamic changes in indole-3-acetic acid (IAA), gibberellic acid (GA_3_), abscisic acid (ABA) and cytokinin zeatin riboside (ZR) concentrations in four different developmental stages all displayed a unimodal type. The concentration of IAA, GA_3_ and ZR initially gradually increased and then gradually decreased after the G3M stage. In contrast, the ABA concentration decreased initially and then gradually increased after the G2M stage. However, the changes of GA_3_, ZR and ABA concentrations in three different developmental internodes were basically inconsistent with the above mentioned results, unlike IAA (Figure 1). Substantial changes in cellular activities and hormone levels involved in these growth stages prompted us to investigate the molecular processes at the transcriptome level.

### Analysis of transcriptome sequencing

#### *Trinity assembly of paired-end sequencing reads*

RNA of different types of culms (including four developmental stages and three portions) was pooled to provide a broad gene library associated with internode elongation. A total of 45.9 million reads with length of 180 bp were obtained in the transcriptome sequencing and 154,903 contigs with an average length of 259 bp were obtained. Through *trinity* assembly, we obtained 60,393 unigenes with an average length of 612 bp and a total length of 36.96 Mbp. Of these, we gained 48,888 unigenes (80.95%) with length 200–900 bp, 9248 unigenes (15.31%) with length 1000–2000 bp, and 2257 unigenes (3.74%) with length > 2000 bp (Table 1; Additional file 1: Figure S1). Sequencing reads were realigned to the unigenes using Trinity [35], allowing up to 2 bp mismatches: about 58.82, 18.16 or 2.01% of the unigenes were realigned by more than 10, 100 or 1000 reads, respectively (Additional file 1: Figure S1).

**Table 1:** Length distribution of contigs, scaffolds and unigenes

**Nucleotides length (bp)**	**Contigs**	**Unigenes**
100-200	100,377	0
201-300	23,766	21,962
301-400	10,119	10,239
401-500	5,311	5,811
501-1000	9,732	12,482
1101-1500	3,237	5,041
1501-2000	1,400	2,601
2001-3000	781	1,735
> = 3000	180	502
Total	154,903	60,393
Minimum length (bp)	100	200
Maximum length (bp)	6,428	8,500
N50 (bp)	332	879
Average length (bp)	259	612
Total Nucloetides length (bp)	40,069,806	36,959,169

#### *Functional classification by gene ontology (GO) and cluster of orthologous groups (COG)*

Out of 60,393 unigenes, 42,127 (69.75%) showed significant similarity to known proteins in the Nr database and matched 27,408 unique protein accessions. However, 24,647 (40.81%) had BLAST hits in the Swiss-Prot database and matched 7,099 unique protein accessions. Altogether, a total of 27,408 unique protein accessions were identified in BLAST searches. This indicated that our study produced a large fraction of Moso bamboo genes. Analysis of 60,393 non-redundant unigenes using BLASTX and ESTscan software revealed that about 42,483 had reliable coding sequences, and the length of most was > 200 aa when translated into proteins. Comparison with the Nr and Swiss-Prot databases revealed that 42,280 unigenes had good comparability with known gene sequences in existing species.

Assignment of GO terms to 32,064 unigenes revealed 209,737 functional terms. Of them, assignments to the cellular component were the majority (91,142, 43.46%), followed by biological processes (79,250, 37.79%) and molecular function (39,345, 18.76%). Under the cellular component category, cell (23,125 unigenes, 25.37%) and organelles (19,280 unigenes, 21.15%) were notably represented. Under the category of biological processes, cellular processes (17,170 unigenes, 21.67%) and metabolic processes (16,813, 21.22%) were the majority. In the molecular function category, 17,710 and 15,853 unigenes were linked to binding and catalytic activity, respectively; it is worthy of mention that a few genes were assigned to nutrient reservoir activity, metallochaperone activity, channel regulator activity and protein tag (Additional file 2: Figure S2). These indicated that some important cell activities, biological, cellular and metabolic processes occurred in culms of developing Moso bamboo shoot.

Of 42,127 Nr hits, 13,957 unigenes were assigned to the COG classifications. Of all 25 COG categories, the largest group was the cluster for general function prediction only (4720, 33.82%); followed by transcription (3300, 23.64%); replication, recombination and repair (2694, 19.30%); posttranslational modification, protein turnover and chaperones (2341, 16.77%); signal transduction mechanisms (2340, 16.77%); translation, ribosomal structure and biogenesis (2284, 16.36%); and carbohydrate transport and metabolism (1975, 14.15%). Only a few unigenes were assigned to nuclear structure and extracellular structure. Furthermore, 614 sequences were assigned to secondary metabolite biosynthesis, transport and catabolism (Additional file 2: Figure S2).

#### *Functional classification by Kyoto encyclopedia of genes and genomes (KEGG)*

The KEGG Pathway database can help to further determine biological functions and interactions of genes. Based on a comparison against the KEGG database using BLASTx with *E* < 10^–5^, of the 60,393 unigenes, 23,897 (39.57%) had significant matches and were assigned to 128 KEGG pathways (Additional file 3: Table S1). Among them, 5,826 (24.38%) unigenes with enzyme commission (EC) numbers were assigned to the metabolic pathways group. Of the unigenes, 1,982 (8.29%), 1,684 (7.05%), 1,652 (6.91%) and 1,627 (6.81%) were linked to the biosynthesis of secondary metabolites, endocytosis, RNA transport and glycerophospholipid metabolism, respectively.

### Digital gene expression (DGE) analysis in rapidly growing culms of bamboo

#### *Tag identification and quantification*

A total of 4.2 million raw tags of the mRNAs extracted from Moso bamboo culms were identified by base calling [36,37]. Approximately 0.12, 0.11, 0.14, 0.12, 0.14 and 0.12 million high quality non-redundant clean tags were obtained in G1M–G4M, G3T and G3B groups (Additional file 4: Figure S3), respectively. Gene annotation was performed by tag mapping analysis using the 60,393 non-redundant unigenes from RNA-seq-based transcriptome analysis as the reference transcript database. Results showed that 45.34, 50.76, 45.17, 46.12, 45.04 and 52.42% of all distinct tags within different group could be mapped to the entire reference database (sense or anti-sense), respectively (Additional file 5: Table S2). The detected sense and anti-sense strands, sense or anti-sense strands mapped by the tags among groups were different (Additional file 4: Figure S3). Among the detectable expressed unigenes, 20,849 had successful annotations.

#### *Depth of sampling*

Saturation analysis can be used to check whether the number of detected genes continues to increase when sequencing amount (total tag number) increases. When the sequencing amount reached 4 M, the number of detected genes almost ceased increasing (Additional file 6: Figure S4) in G1M–G4M, G3T and G3B libraries.

#### *Comparison of gene expression level between libraries*

**Figure 2:**
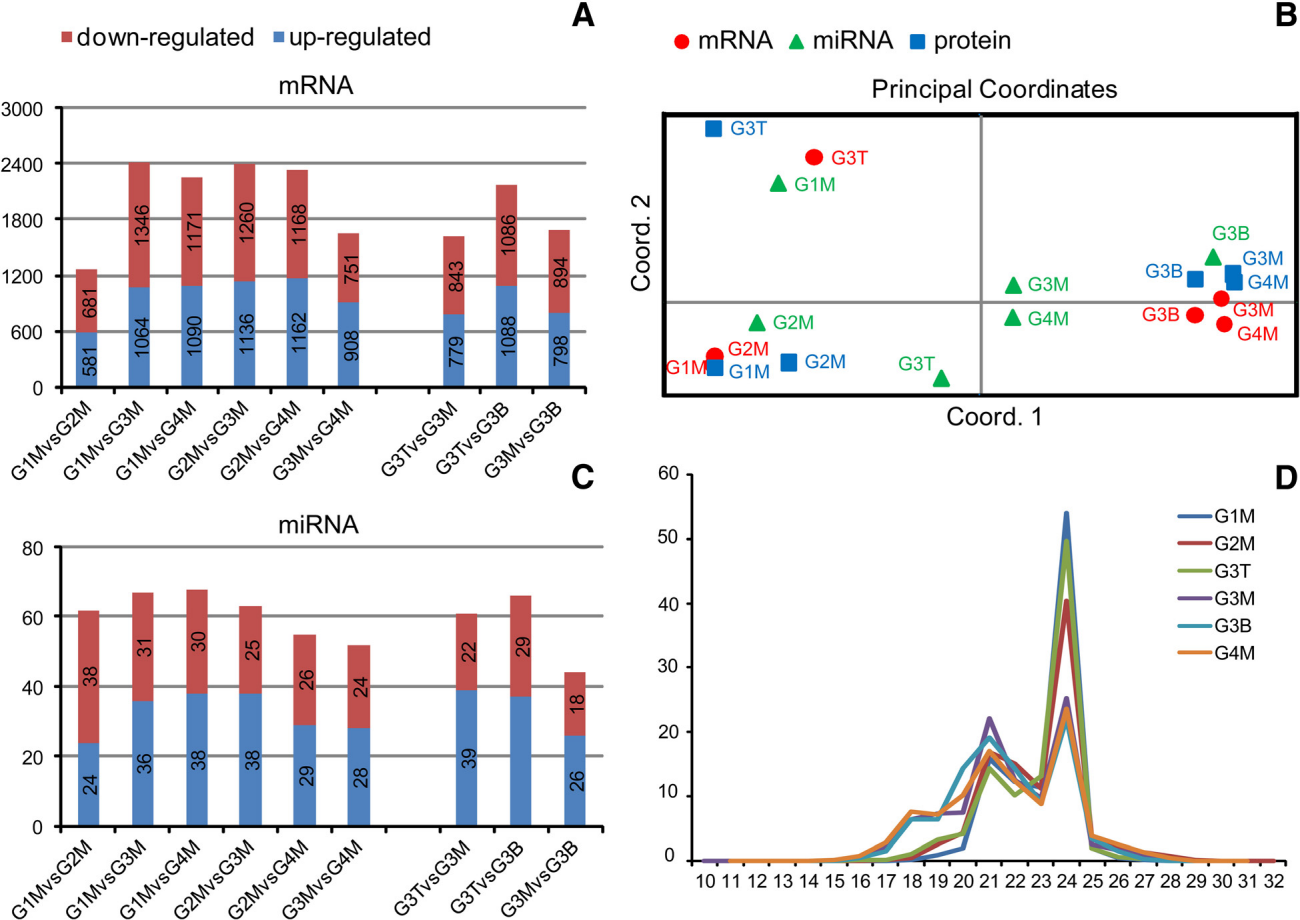
**The histogram showing the number of differentially expressed genes (A) and miRNAs (B) between libraries. C** represents the length distribution of the small RNA genes in different libraries. **D** represents Principal Coordinates Analysis (PCA) of differentially expressed genes, miRNAs and proteins in different libraries. PCA indicated that six samples were divided into two groups, one was G1M, G2M and G3T, the other was G3M, G4M and G3B. G1M, G2M, G3M and G4M represent four developmental stages in turn. G3T, G3M and G3B represent top, middle and basal internode of the third developmental stage, respectively. The red circle represent mRNAs, green triangle represent miRNAs, and blue square represent proteins.

The differences in tag frequencies in the G1M–G4M, G3T and G3B libraries were used to estimate gene expression levels at different development stages or different internodes. The black and white histograms represent transcripts up-regulated or down-regulated in number by more than twofold in every library, respectively. For developmental stages, the highest amount of differential genes (2,410) was detected between G1M and G3M, and the least (1,262) between G1M and G2M. For developmental positions, the highest were observed between G3T and G3B (2,174), followed by G3M vs G3B (1,692), and G3T vs G3M (1,622). The number of down-regulated genes was more than that of up-regulated genes with the exception of G4M vs G3M (Figure 2A). Moreover, 5837 genes that met our threshold criteria for at least one of the 15 DGE comparisons were clustered using principal coordinate analysis (PCA) and hierarchical clustering. Six samples were separated into two groups, one was G1M, G2M and G3T group, and another was G3M, G4M and G3B group (Figure 2B). The result was consistent with that of hierarchical clustering (Additional file 7: Figure S5).

#### *Expression profiles of mRNAs during culms development*

**Figure 3:**
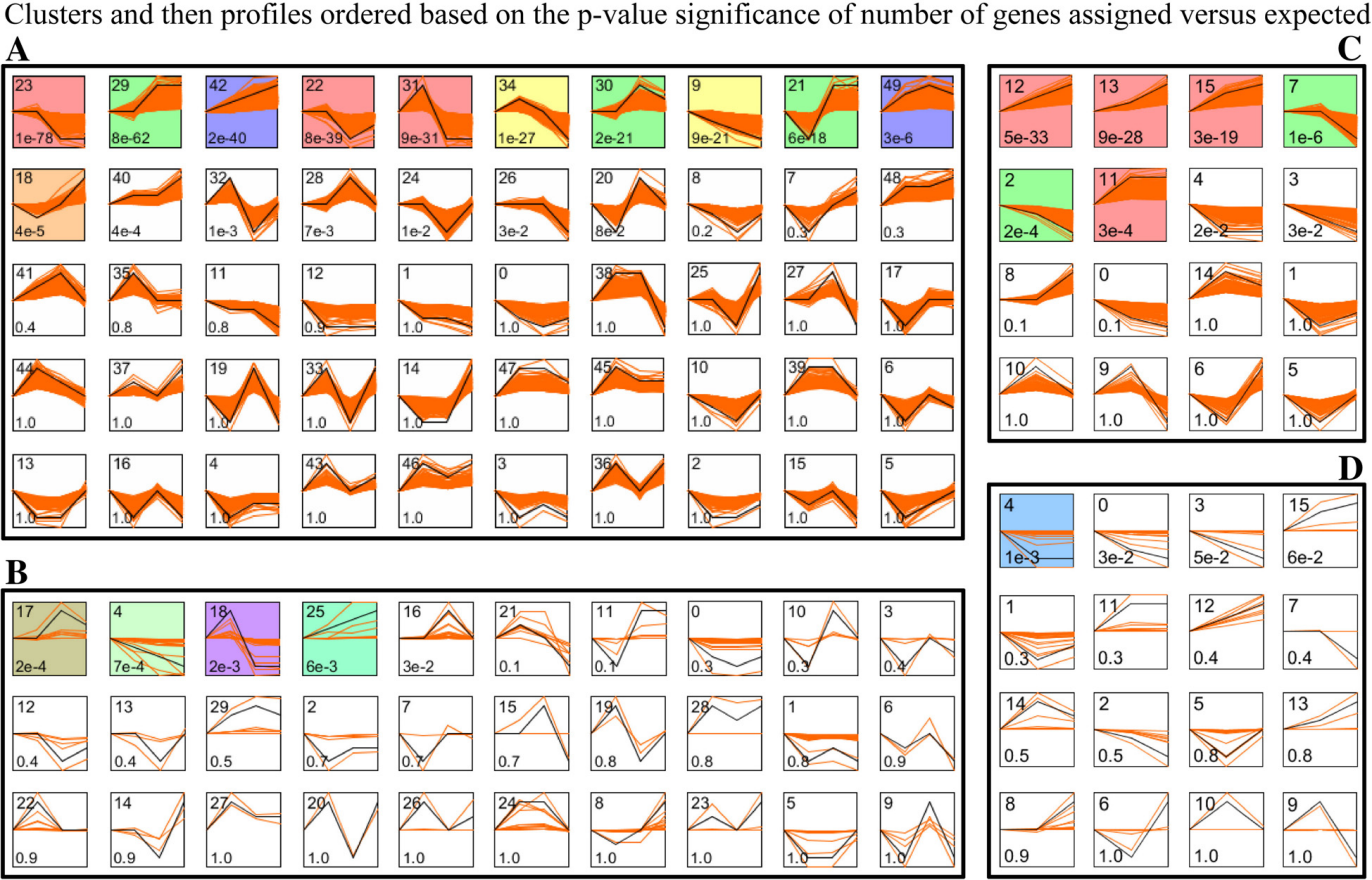
**Short Time-series Expression Miner clusters of expression profiles.** The profiles ordered based on the p-value significance of number (at bottom-left corner) of genes or miRNAs assigned versus expected. Colour square frame denote significant profiles (P-value ≤ 0.01). Each graph displays the mean pattern of expression (black lines) of the profile genes. The number of profiles in each cluster is at the top left corner of each STEM. **A** and **B** represent the development-specific mRNA and miRNA profiles, respectively. **C** and **D** represent the internode-specific mRNA and miRNA profiles, respectively. The x-axis represents time (development-specific or internode-specific stages) and the *y-*axis represents log_2_ fold change of gene expression.

The 5,837 genes were further clustered using Short Time-series Expression Miner (STEM) [38]. It identified 11 (1,024 genes) or 6 (923 genes) significant gene profiles (P-Value ≤ 0.01) in development or internode, respectively (Figure 3A and 3C; Table 2; Additional file 8: Table S3).

**Table 2:** Genes and miRNAs grouped into significant cluster profiles by STEM analysis

**Profile model**	**Num. Genes Expected**	**Num. Genes Assigned**	**P-Value**
**mRNA**			
**Development-specific genes**			
Profile 9 (0,-1,-2,-3)	177	85.7	1.50E-18
Profile 42 (0,1,2,3)	177	85.7	1.50E-18
Profile 18 (0,-1,0,2)	109.5	74.1	4.90E-05
Profile 34 (0,1,0,-2)	164	89.5	5.70E-13
Profile 21 (0,-1,1,1)	157	95.6	3.40E-09
Profile 31 (0,1,-1,-1)	179	85.3	2.10E-19
Profile 22 (0,0,-2,-1)	251	95.6	3.50E-41
Profile 30 (0,0,2,1)	148	85.3	3.00E-10
Profile 23 (0,0,-1,-1)	371	134.8	5.70E-66
Profile 29 (0,0,1,1)	298	134.8	2.00E-35
Profile 40 (0,1,1,3)	94.5	57.6	3.40E-06
**Internode-specific genes**			
Profile 2 (0,-1,-3)	171	128.8	1.60E-04
Profile 12 (0,1,2)	226	92.7	4.70E-33
Profile 13 (0,1,3)	237	108.8	8.90E-28
Profile 15 (0,2,3)	239	128.8	3.10E-19
Profile 7 (0,0,-1)	304	231.7	1.20E-06
Profile 11 (0,1,1)	284	231.7	2.80E-04
**miRNA**			
**Development-specific miRNAs**			
Profile model	Num. miRNA Expected	Num. miRNA Assigned	P-Value
Profile 4 (0,-1,-2,-3)	27	13.5	6.50E-04
Profile 17 (0,0,2,1)	28	13.3	2.40E-04
Profile 18 (0,1,-1,-1)	25	13.3	2.40E-03
Profile 25 (0,1,2,3)	24	13.5	5.50E-03
**Internode-specific miRNAs**			
Profile 4 (0,-1,-1)	130	101.3	1.20E-03

The development-specific gene clusters, STEM profile 9 (177 genes) and profile 42 (177 genes) showed respectively down and up regulated along with the culm development and consistent with the decrease of cell nuclei number and prolong of cell length (cell division) along with clum development and aging. STEM profile 18 (110 genes) and 34 (164 genes) have a reversal in the trend of expression change between development and aging, and profile 18 was consistent with the change of ABA content. Profile 21 (157 genes) and Profile 31 (179 genes) gene clusters have a reversal in G2M stage and no change at later stage, while profile 22 (251 genes) and Profile 30 (148 genes) gene clusters have a reversal in G3M stage and no change at earlier stage. Profile 23 (371 genes) and Profile 29 (298 genes) show up- or down-regulated during the middle stage, and no change at the earlier and later stages. Contrary to profile 23 and 29, Profile 40 contained 95 genes (Figure 3A; Additional file 8: Table S3).

The internode-specific gene clusters, Profile 2 (171 genes) and 7 (304 genes) represented down-regulated genes, were consistent with the decrease of cell nuclei (cell division) along with internode development. Profile 11 (284 genes), 12 (226 genes), 13 (237 genes) and 15 (239 genes), up-regulation internode-specific genes, were consistent with the prolong of cell length (cell division) along with internode development (Figure 3C; Additional file 8: Table S3).

### miRNAs regulate expression changes in rapidly growing culms of bamboo

#### *Sequencing and data analysis*

To investigate the miRNA component of small RNAs and the dynamic changes of the miRNAs during the rapid growth of developing culms in Moso bamboo, we purified the culms of G1M-G4M, G3T and G3B from bamboo and sequenced their small RNAs using Solexa high-throughput technology. After removing sequences of low quality, 5′ adaptor contaminants, 3′ adaptor null, insert null, poly A and RNAs smaller than 18nt, we obtained 12,724,584, 14,638,070, 13,279,680, 12,065,802, 12,841,021 and 12,360,293 clean reads, respectively (Additional file 9: Table S4). These high-quality small RNAs were used for further analysis.

Of the millions of high-quality small RNAs from the individual libraries, 72.21–95.81% was 20 to 24 nucleotides in length, which is the typical size range for Dicer-derived products. The major component of small RNAs in all libraries was 24 nucleotides long. Throughout clum development, the proportion of 24-nucleotide small RNAs decreased and the 21-nucleotide population increased in G1M-G4M, while G3M contained mostly 21-nucleotide small RNAs (Figure 2D).

#### *Prediction of novel miRNA and comparison of miRNA expression level between libraries*

In total, we obtained 732 miRNAs and 453 predicted novel miRNAs. Of these novel miRNAs, 26, 61, 101, 57, were expressed in G1M-G4M, and 92 in G3T, 116 in G3B, respectively (Additional file 10: Table S5). Differ from the result of differentially expressed genes; the number of differential miRNAs in the initial stages was more than that in the descendent stages, the amount of up-regulated miRNAs was more than that of down-regulated miRNAs with the exception of G1M vs G2M (Figure 2B). However, PCA and hierarchical clustering showed the consistency between miRNAs (165 miRNAs) and mRNAs (5837 genes), that is, six samples were separated into two groups, one was G1M, G2M and G3T group, another was G3M, G4M and G3B group, just as protein (213 proteins) clustering result (Figure 2C; Additional file 7: Figure S5). Furthermore, compared with miRNA expression, higher consistency between mRNAs and proteins was observed.

#### *Expression profiles of miRNAs during culms development*

To directly compare the expression patterns of these miRNAs in the different development stages or internodes, four development-specific profiles (54 miRNAs) or one internode-specific profile (101 miRNAs) were identified significantly by STEM cluster, respectively (Figure 3B and 3D and 3D; Table 2; Additional file 8: Table S3). Profile 4 (down-regulation, 27 miRNAs) and 25 (up-regulation, 24 miRNAs) continue the development trend. Profile 17 (28 miRNAs) and 18 (25 miRNAs) have a reversal in the trend of expression change between development (Figure 3B). The internode-specific significant miRNAs cluster, profile 4 (down-regulation) contained 130 miRNAs (Figure 3D).

### The integrated analysis between STEM and positive/negative correlation

In general, the mature miRNA negatively regulates their target mRNAs by either translational repression or mRNA degradation[39]. Through target prediction using the Miranda web service, we found 6598 targets for 671 known-miRNAs and 4616 targets for 298 novel-miRNAs (Additional file 11: Table S6). Analysis to identify negative correlations between miRNA and mRNA expression was done using an in house R script, and pearson correlation coefficient (cor) was computed for each miRNA and their predicted target mRNAs. Forty-five of 121 miRNA-mRNA pairs combined by 37 unique miRNAs and their 44 target mRNAs presented an anti-correlationship between the expression level of miRNAs and mRNAs (cor < −0.81 and P-value of correlation ≤0.05) (Additional file 12: Table S7). Positive correlation between 2-DE staining intensity and gene expression levels determined by RNA-Seq for fast-growing clum in Moso bamboo are summarized in Additional file 12: Table S7. Statistical pearson correlation ranged from 0.53–0.93, and eight of 25 mRNA-protein pairs combined by 24 unique corresponding proteins presented a plus correlationship (cor > 0.81 and P-value of correlation ≤0.05).

**Figure 4:**
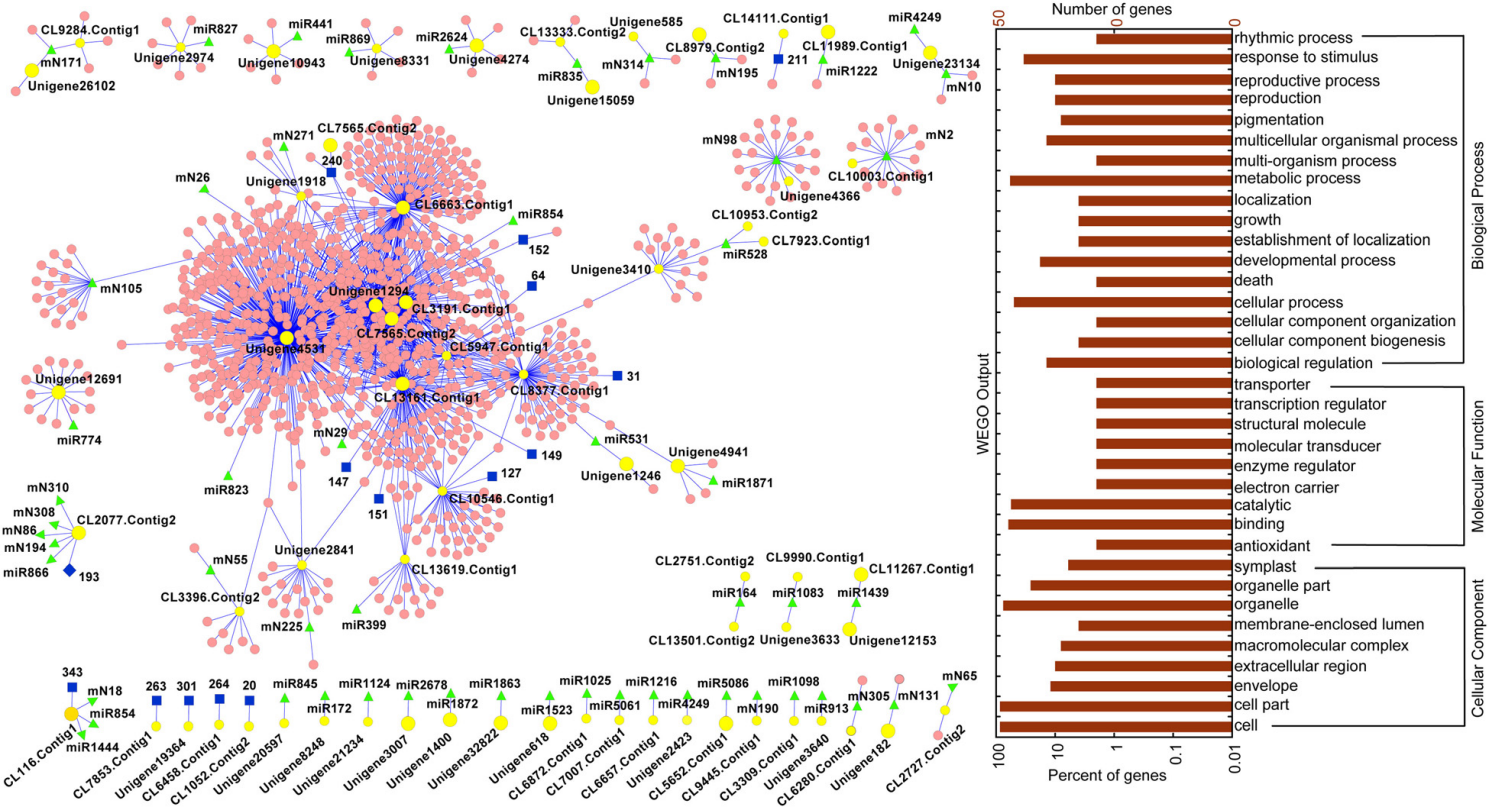
**A network diagram based on gene interaction, STEM cluster and correlation between mRNA-protein and miRNA-mRNA (left).** The negative correlation analysis depend on miRNAs and microRNA-regulated target genes, genes and their encode proteins carry out positive analysis. Gene-gene interaction was compared with rice gene interactions. Red circle represent genes, yellow circle represent 64 target genes, 27 statistically significant genes were labeled in bigger yellow circles, blue square represent proteins, green triangle represent miRNAs. Gene Ontology classification of assembled unigenes (right). Fifty of 64 unigenes with BLAST matches to known proteins were assigned to three main categories: biological process, cellular component and molecular function.

Based on the analysis of intersection/integration between significant STEM and positive/negative correlation, seventy three pairs were obtained, including 64 genes, 55 miRNAs (34 known- and 21 novel-miRNAs) and 15 proteins. Of the 73 pairs, 34 pairs (27 genes) were significant correlation (P-value of correlation ≤0.05) (Additional file 12: Table S7). According to interaction of 64 genes and correlation between miRNA-mRNA and mRNA-protein, a complex network was built in Figure 4, and the 64 genes were in the core position (yellow circle). The GO annotation of 64 genes involved in grow and reproduction (especially xylem or phloem histogenesis and formation, leaf morphogenesis), auxin and ABA mediated signaling, cellular process (ribosome biogenesis and cell division or death), metabolic process (protein ubiquitination, photorespiration, glycolysis, proteolysis, glycan and energy metabolism, especially hemicellulose metabolic process), biological regulation (such as regulation of ARF GTPase activity, regulation of anthocyanin biosynthetic process), and response to stimulus (response to cold or cadmium ion). The pathways included metabolism (carbohydrate metabolism, lipid metabolism, nucleotide metabolism, amino acid metabolism, energy metabolism, glycan biosynthesis and metabolism, metabolism of terpenoids and polyketides, biosynthesis of other secondary metabolites), genetic information processing (translation), cellular processes (transport and catabolism), environmental information processing (signal transduction, membrane transport) and environmental adaptation (plant circadian rhythm) (Additional file 12: Table S7).

## Discussion

Here, we report on the first next-generation sequencing study of the temporal and spatial mRNA and miRNA expression profiling of bamboo internodes. A DGE approach enabled identifying those mechanisms related to rapid growth of developing culms in Moso bamboo. STEM, positive and negative correlation analysis were perfectly applied in integrated analysis of multi-omics data. These provided a molecular basis underlying the phenomenon of sequentially elongated internodes from the base to the top.

### Temporal and spatial growth patterns of internodes in moso bamboo

The initial, ascending, boosting and terminal stages are involved in growth phases in bamboo culms. Sequentially elongated internodes from the base to the top in bamboo have been reported [14]. In our previous study, the growth stages of different internodes within the same culm were established using morphological observations, endogenous hormones and anatomical structure analysis [29]. However, the molecular basis underlying this phenomenon remains unclear. To determine transcriptional and post-transcriptional regulation changes that occurred during different growth stages of bamboo culms, we analyzed six internodes from different stages (G1M–G4M) or different portions (G3B, G3M and G3T) of the same culm (at the G3M stage). It is generally recognized that different numbers of differentially expressed genes could represent or describe different growth degrees, i.e. the greater the number between stages, the larger the difference between them. Comparisons of gene expression level between the six DGE libraries indicated that the relationship between G1M and G2M was the closest and the farthest between G2M and G3M for four developmental stages. However, for the three spatial positions the relationship between G3T and G3B was the farthest, followed by G3M and G3B, and then G3T and G3M. In addition, PCA and hierarchical clustering analysis based on differentially expressed genes showed that G1M, G2M and G3T were classified in the one group; whereas G3M, G4M and G3B were classified in another group, and cells of the latter were longer than the former. This was consistent with the clustering result from differentially expressed proteins and similar to differentially expressed miRNAs, indicating the consistency in the differentially expressed levels of transcripts, miRNAs and proteins among stages. All our data suggested that G1M, G2M and G3T were involved in the initial or ascending stages; and G3M, G4M and G3B were involved in the boosting or descendent stages. This result was not surprising, because although the whole plants were defined as a specific growth stage (usually defined according to the middle internode), different portions of the same plant may be at different stages resulting from the sequential basal–apical elongation of the culm internodes[14]. When the growth of the whole plant was in boosting stage (G3M), the growth of the base internode (G3B) might be in descendent stage, while the top internode (G3T) might be in the initial or ascending stage. Thus, our study provided a molecular basis underlying the phenomenon of sequentially elongated internodes from the base to the top.

### GO and pathways involved in rapidly elongating culms of *P. Heterocycla*

**Figure 5:**
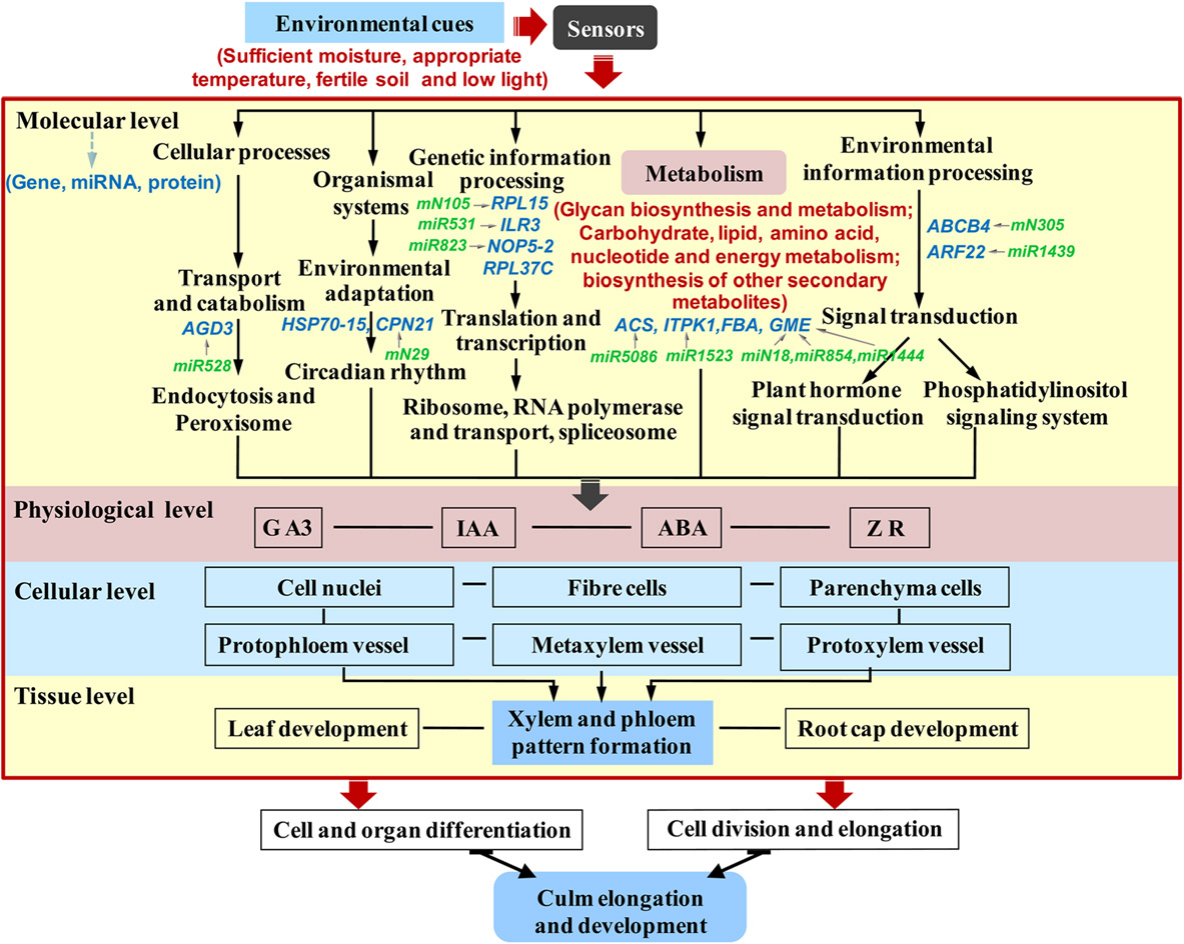
**A scenario of bamboo’ culm elongation and development at molecular, physiological, cellular and tissue level.** During the growth season, some environmental cues such as sufficient moisture, appropriate temperature, fertile soil and low light drive plant circadian rhythm, stimulate cell differentiation, division and growth, and biosynthesis of plant hormones, especially auxin and ABA, through some signaling sensor and transduction pathways. And then the cells take up energy or nutrients through glycolysis, photorespiration, glycan and energy metabolism to maintain normal metabolic activity, which promotes cell division, elongation or death, lignin and cellulose deposition, eventually leading to elongation and development of the Moso bamboo’ culm.

Short Time-series expression data/experiments provide a wealth of information regarding the global view of the dynamic networks that are activated in dynamic biological processes [38,40]. Many studies show that a comprehensive understanding of the control of gene expression will require quantitative information at all levels, from DNA variants through to differential stability of the products or their regulative factors [41-43]. Our temporal or static omics data, including transcriptome, post-transcriptome and translatome/proteome, play an even bigger part in molecular mechanisms study of dynamic bamboo’ culm development. By integrating such data with their positive/negative correlation analysis data sets we obtain the complete set of gene/protein or miRNA that are activated, their dynamics and interactions, the role that different gene/protein or miRNA have in the process and the differences in processes within and between different growth stages. At last the core genes focused on 64 genes and their correlative 55 miRNAs and 15 proteins were obtained, the GO and pathway analysis revealed that genes involved in response to stimulus, cell cycle, cellular component, regulation of plant hormone levels, many metabolism and signal transduction played major roles in persistently elongating culms of *P. heterocycla*. Meanwhile, with the development of bamboo culm, gradually declined cell nuclei, gradually elongated parenchyma and fiber cells, dynamic changes of a unimodal-type endogenous hormones concentration were observed in our study. According to these, we proposed some scenarios involved in culm elongation (Figure 5). During the growth season, some environmental cues such as sufficient moisture, appropriate temperature, fertile soil and low light drive plant circadian rhythm, stimulate cell differentiation, division and growth, and biosynthesis of plant hormones, especially auxin and ABA, through some signaling sensor and transduction pathways. Furthermore, the cells take up energy or nutrients, possibly supplied by the mother bamboo rhizome and root system, in the cellular path through glycolysis, photorespiration, glycan and energy metabolism to maintain normal metabolic activity, which promotes cell division, elongation or death, lignin and cellulose deposition, eventually leading to growth of the culm.

Stimulus response of an organism is usually due to some external condition or event. Many genes/proteins involved in response to stimulus have been reported in the developing maize rachis [44] and fruit development of sweet orange [45]. Seventeen unigenes (Additional file 12: Table S7) involved in response to stimulus, such as carbohydrate, cytokinin, auxin or ABA stimulus, cold, blue light, cadmium ion, gravitropism were detected during the fast-growing stage of bamboo, indicating the important role of environmental cues in bamboos’ rapid growth. Signal transduction occurs when an extracellular signaling molecule activates a cell surface receptor. In turn, this receptor will alter intracellular molecules with a response. The hormone-mediated signaling pathway was one signal transduction found to be regulated in fast-growing culms of bamboo. It comprised auxin mediated signaling pathway (one unigene) and abscisic acid mediated signaling pathway (one unigene). The data indicated some genes in diverse hormone (auxin, ABA, ethylene and GAs) signaling pathways are regulated in different trends or stages in rice seed development [46]. The phosphatidylinositol signaling system was also found to be regulated in fast-growing culms of bamboo. It comprised inositol or phosphatidylinositol kinase activity (one unigenes) and nucleoside-triphosphatase activity (five unigenes). Some studies also illustrated that the phosphatidylinositol signaling system played an important role in plant development, such as storage root formation [47], maize growth [48] and rice seedling germination [49]. Our study will help to expand knowledge of the function of the phosphatidylinositol signaling system in plant growth.

Plant hormones are major regulators of plant growth and development. Endogenous hormones as well as environmental factors can regulate progression through the cell cycle: shaping the plant and affecting seed growth, time of flowering, sex of flowers, and senescence of leaves and fruit [50,51]. Auxins, the first identified plant hormones (and commonly IAA), are compounds that positively influence cell enlargement, bud formation and root initiation. They affect cell elongation by altering cell wall plasticity. They stimulate the cambium (a subtype of meristem cells) to divide, and in stems cause secondary xylem to differentiate. Some studies have shown that auxin promotes proliferation activities in the cambium during secondary growth. For example, it was indicated that auxin maxima are established and genes associated with auxin signaling are up-regulated in the actively proliferating cambial cells in hybrid aspen [52]. By contrast, general reduction in polar auxin transport and down-regulation of auxin signaling genes were observed during the phase of cambium dormancy [52,53]. Consistently, disruption in auxin signaling/responsiveness led to detrimental effects on cambial activities, reducing secondary growth in hybrid aspen plants [54,55]. Similarly, several other plant hormones, in particular cytokinin, ethylene and gibberellins are known to regulate cambial development [56,57]. In our studies, four major endogenous hormones (IAA, GA_3_, ZR and ABA) appeared to strongly influence the cell elongation phase. In the present study, four unigenes involved in auxin efflux and abscisic acid mediated signaling pathway, ethylene biosynthetic process, response to cytokinin stimulus, were identified (Additional file 12: Table S7). This indicates that in the bamboo growth season, environmental cues might stimulate cell division, growth or death through regulating hormone transport of influencing the expression of hormone response factors.

A cellular component may be an anatomical structure (e.g. rough endoplasmic reticulum or nucleus) or a gene product group (e.g. ribosome, proteasome or protein dimer). During secondary vascular development in poplar, the majority of the integral proteins identified were plasma membrane proteins [58]. Some studies have suggested that up-regulation of plasma membrane aquaporins improves the photosynthetic activity and growth of Eucalyptus trees [59]. In our study, numerous cell nuclei were found in parenchyma and fiber cells – an obvious sign of cell division – indicating the presence of meristematic tissue. As the culm developed, the number of nuclei declined, until there were almost no detectable nuclei during the late stages of development. Meanwhile, the length of parenchyma and fiber cells increased continuously during development [29]. Many genes involved with the plasma membrane (12 unigenes), organelles (38 unigenes) such as the nucleolus (two unigenes), organelle envelope (six unigenes), chloroplast thylakoid membrane (two unigenes), cell wall (three unigenes), and Golgi membrane (one unigenes), cytosol (10 genes) and apoplast (five genes) influenced the fast growth of the culm (Additional file 12: Table S7). Most of them peaked at the ascending or boosting stages. This suggests that cell division is essential to rapid elongation of the culms during their early developmental stages. Remarkably, six genes involving in biosynthesis of lignin and cellulose/hemicellulose, the major cell wall components, were detected, it suggests that lignocellulose is accumulated along with the development of bamboo’ culm.

Metabolic processes are necessary for life. A number of excellent studies have indicated that metabolic processes were among the most regulated during developing stages of plants, and these genes were involved in many biological processes: e.g. glycan biosynthesis and metabolism, and the metabolisms of carbohydrates, lipids, amino acids and energy [26,44,45,60,61]. In developing bamboo culm in the present study, the dominant metabolic processes (27 unigenes) included glycan biosynthesis and metabolism, the metabolisms of carbohydrates, lipids, amino acids, nucleotides, energy and biosynthesis of other secondary metabolites (Additional file 12: Table S7). These metabolic processes can provide the energy and components for DNA replication, signal transduction, cellular growth, hormone biosynthesis, cellulose and lignin biosynthesis, which are essential for the rapid culm elongation under conditions of light deficiency.

## Conclusions

To the best of our knowledge, this study is the first exploration to characterize the temporal and spatial transcriptome, miRNA and mRNA expression profiling in a developing bamboo culm. We provided a molecular basis underlying the phenomenon of sequentially elongated internodes from the base to the top. Several key pathways such as environmental adaptation, signal transduction, translation, transport and many metabolism, and GO terms such as hormone-mediated signaling, cell growth, division and differentiation, primary shoot apical meristem specification, xylem and phloem pattern formation, protein modification, response to stimuli, metabolic process and biological regulation were identified as involved in rapid growth of bamboo culms. In addition to gaining more insight into the unique growth characteristics of bamboo, we provide a good case study to analyze mRNA and miRNA expression of a non-model plant species using high-throughput short-read sequencing. Cluster concordance of protein, transcript and post-transcriptional levels are obvious. Above all, we demonstrate that the integrated analysis of our multi-omics data, including transcriptome, post-transcriptome/miRNAs and translatome/proteome, yield more complete representations and additional biological insights, especially the complex dynamic processes occurring in the rapid growth of developing culms in Moso bamboo.

## Methods

### Plant materials

Culm samples from a natural population of *P. heterocycla* were harvested in spring 2009 at the Dagangshan Forest Ecosystem Research Station in Jiangxi Province of China (27°30′–27°50′N, 114°30′–114°45′E), where *P. heterocycla* is typically distributed in the subtropical zone. To ensure that there were absolute minimal differences in the growth conditions, all culms were sampled within the distribution of 50 × 50 m^2^. To ensure that equivalent staged samples and developmental stages were taken as replicates, individual plants were sampled with the same height at each stage. Based on previous observations [14,29] and our hormonal and anatomical results, four developmental stages or three developmental internodes were defined according to the different unearthed heights of individual plants (0.05, 1.00, 6.00 or 12.00 m; nominated as G1M–G4M, respectively) or different portions (basal internode, G3B; middle internode, G3M; and top internode, G3T) of the same culm (at the G3M stage), respectively. The samples, each with three biological replicates (three trees), were cut from tissue inside the epidermis located in the basal part of each middle internode for different stages or for different internodes at the same stage as mentioned above. The samples were immediately frozen in liquid nitrogen and kept at −80°C until analysis.

### Light microscopy and ELISA

Approximately 0.5-cm^3^ samples of culm tissue were fixed in FAA (Formalin-Acetic-Alcohol) buffer and exhausted with an aspirator pump. Subsequently, serial transverse and longitudinal sections (8 μm thick) from paraffin-embedded tissue were sequentially stained with safranin and fast green. Finally, these sections were observed with a Zeiss Axiophot light microscope (Zeiss, Jena, Germany). Digital images were analyzed using Zeiss Axio Vision 4.6.

The method for extraction, purification and quantification of endogenous plant hormones IAA, GA_3_, ABA and ZR were modified from the description of Wang [62]. ELISA kits used for estimation of the hormone levels came from China Agricultural University (Beijing, China). One-way ANOVA and least significant difference (LSD) tests were used to check for differences in cell morphology and hormone content. *P* ≤ 0.05 was considered statistically significant.

### Sample preparation for RNA-seq and transcriptome analysis

Tissue samples from 18 culms (six development stages or internodes, each with three biological replicates) were collected for RNA preparation. Total RNA was isolated using a TRIzol reagent (Gibco BRL) following the manufacturer’s instructions, and then beads with Oligo (dT) were used to isolate poly (A) mRNA. After fragmentation, random hexamer-primer was used to synthesize the cDNA. Short cDNA fragments were purified and followed by end reparation, adenylation and sequencing adapter ligation. After PCR amplification of the selected fragment, one pooling library was sequenced using Illumina HiSeq™ 2000. The raw sequencing datasets of transcriptome were deposited in the Sequence Read Archive (SRA) of NCBI (http://www.ncbi.nlm.nih.gov/sra) under accession number SRX329521.

Transcriptome *de novo* assembly was carried out with the short reads assembling program – Trinity [63] : it first combined reads with certain length of overlap to form contigs, and then reads are mapped back to contigs; with paired-end reads, it is able to detect contigs from the same transcript as well as the distances between these contigs. Finally, Trinity connects the contigs, and gets sequences that cannot be extended on either end. Such sequences are defined as Unigenes. After clustering, the unigenes will be divided to two classes. One is clusters with the prefix CL, and another is singletons with the prefix Unigene. Finally, BLASTx alignment (*E* < 10^–5^) between unigenes and protein databases such as non-redundant protein (Nr), Nucleotide (Nt), Swiss-Prot and KEGG, then COG was performed, and the best aligning results used to determine sequence direction of unigenes. When a unigene was not aligned in any of the above databases, ESTScan [64] was used to predict its coding regions and sequence direction. On the basis of Nr annotation, the Blast2GO and the WEGO program were used to perform GO functional classification. COG and the KEGG Pathway database were also used to further determine the biological functions and interactions of unigenes.

### DGE-tag profiling

DGE sample preparation was performed using Illumina Gene Expression Sample Prep Kit. Of total RNA, 6 μg was purified using Oligo (dT) magnetic beads, and then Oligo (dT) was used as a primer to synthesize the first and second-strand cDNA. The 5′-ends of tags were generated by *NlaIII*. The fragments apart from the 3′-cDNA fragments connected to Oligo (dT) beads were washed away and the Illumina adaptor 1 was ligated to the sticky 5′-end of the digested bead-bound cDNA fragments. The junction of Illumina adaptor 1 and CATG site was cut by *MmeI*. After removing 3′-fragments by magnetic bead precipitation, Illumina adaptor 2 was ligated to the 3′-ends of tags, acquiring tags with different adaptors of both ends to form a tag library. After 15 cycles of linear PCR amplification, purification by 6% TBE PAGE gel electrophoresis, and denaturation, the single-chain molecules were fixed onto the Illumina Sequencing Chip (flowcell). After cluster generation, sequencing with the method of sequencing by synthesis was performed using Illumina HiSeq™ 2000. The raw reads were submitted to the SRA of NCBI under the accession number SRX329613.

### Aligning DGE tags to the reference transcriptome data set and identification of differentially expressed genes

Clean tags and count number of six DGE libraries from G1M–G4M, G3B and G3T groups were collected and summarized using custom Bioperl scripts. All tags were mapped to the reference transcriptome generated by RNA-seq. To monitor mapping events on both strands, both sense and complementary anti-sense sequences were included in the mapping process. Only perfect matches over the entire 21-bp length of the 17-bp tag plus the 4-bp *Nla*III recognition site were allowed. This study was limited to tags that mapped to ORFs (open reading frames) only and cannot show tags that mapped to mRNA with long 3′-UTRs (untranslated regions). Rigorous algorithms were developed to identify differentially expressed genes between two groups [65]. The correlation of the detected count numbers between parallel libraries was statistically assessed using Pearson’s correlation. In addition to the *P*-value, false discovery rate (FDR) was used in DGE to determine the threshold of *P*-value in multiple test and analysis [66]. In this research, *P*-value ≤ 0.01, FDR ≤ 0.001, and the absolute value of log_2_Fold-change ≥1 or ≤ −1 were used as threshold to judge the significance of gene expression differences.

### Small RNAs preparation and analysis

The experiment process of miRNA was described in Hafner et al. [67]. Briefly, low molecular-weight RNA was isolated and sequenced following the Small RNA Sample Preparation Protocol (Illumina). The 50nt sequence tags from HiSeq sequencing will go through the data cleaning first, which includes getting rid of the low quality tags and several kinds of contaminants. Length distribution of clean tags is then summarized. The raw reads are available in the NCBI SRA database under the accession number SRX330460. Afterwards, the standard bioinformatics analysis will annotate the clean tags into different categories and take those which cannot be annotated to any category to predict the novel miRNA. The characteristic hairpin structure of miRNA precursor was used to predict novel miRNA and software Mireap (http://sourceforge.net/projects/mireap/files/mireap/) was developed to predict novel miRNA by exploring the secondary structure, the Dicer cleavage site and the minimum free energy of the unannotated small RNA tags which could be mapped to genome.

### Correlation analysis

Analysis to identify negative correlations between miRNA and mRNA expression was done using an inhouse R script. Briefly, normalized miRNA and mRNA data were sample-matched for all samples with both miRNA and mRNA sequencing data. Then for each miRNA, Pearson correlation coefficients were computed for its predicted target mRNAs, and a contingency table was created for all mRNAs, which was used to assess the enrichment level of the negative correlated mRNAs (correlation < 0 and P-value of correlation ≤ 0.05) within predicted targets of the intended miRNA using Fisher’s exact test. The method of positive correlation analysis between mRNAs and corresponding proteins was the same as that of negative correlations.

## Competing interests

The authors declare that they have no competing interests.

## Authors’ contributions

JGZ designed and supervised the study. CYH designed experiment and analyzed the data as a whole and wrote the manuscript. KC performed proteome analysis. AGD and YFZ coordinated character investigation and collected bamboo samples. All authors read and approved the final manuscript

## Supplementary Material

Additional file 1: Figure S1 Quality and coverage evaluation of assembled unigenes. Distribution of unique-mapped reads of the assembled unigenes. A: Length distribution of unigene. B: Read distribution of unigene. Click here for file

Additional file 2: Figure S2 Gene Ontology (right) and clusters of orthologous groups (COG) (left) classification of assembled unigenes. A total of 32,064 unigenes with BLAST matches to known proteins were assigned to three main categories: biological process, cellular component and molecular function. Out of 42,127 Nr hits, 13,957 sequences were assigned to 25 COG classifications. Click here for file

Additional file 3: Table S1 List of 125 KEGG pathways for unigenes. Click here for file

Additional file 4: Figure S3 Distribution of tags and gene expression among G1M-G4M, G3T and G3B groups. The distribution of tags matches that of gene expression among groups. Furthermore, an increase in tags or gene expression is accompanied by a decrease in the frequencies of tags or genes expression. Click here for file

Additional file 5: Table S2 Summary of tag mapping in DGE analysis for G1M–G4M, G3T and G3B groups. Click here for file

Additional file 6: Figure S4 Sequencing saturation evaluation of G1M-G4M, G3T and G3B libraries. Click here for file

Additional file 7: Figure S5 Hierarchical clustering of differentially expressed genes, miRNAs and proteins. A total of 213 differentially expressed proteins (DEPs) were detected by two-dimensional gel electrophoresis (2-DE) and identified by matrix-assisted laser desorption/ionization time-of-flight/time-of-flight mass spectrometry (MALDI-TOF/TOF MS). G1M, G2M, G3M and G4M represent four developmental stages in turn. G3T, G3M and G3B represent top, middle and basal internode of the third developmental stage, respectively. The color ranges from green to red for the down-regulated and up-regulated genes, respectively. Click here for file

Additional file 8: Table S3 A. The significant STEM clusters of mRNAs. Eleven (1,024 genes) development-specific and six (923 genes) internode-specific gene profiles (P-Value ≤ 0.01) were obtained separately. B: The significant STEM profiles of miRNAs. Four (54 miRNAs) development-specific and one (101 miRNAs) internode-specific miRNA profiles were identified separately. Click here for file

Additional file 9: Table S4 Statistics of small RNA sequences from the individual libraries. Click here for file

Additional file 10: Table S5 The list of know-miRNA and novel-miRNA in each libraries. Click here for file

Additional file 11: Table S6 The list of miRNAs and their predicted target genes. Click here for file

Additional file 12: Table S7 A: The negative correlation pairs between miRNAs and mRNAs, the positive correlation pairs between mRNAs and proteins. B: The statistical data pairs from the integrated analysis of STEM, positive/negative correlation. C: The GO annotation and enrichment of 64 core genes. Click here for file
