# Supplementary figures and images for: Next-generation sequencing-based mRNA and microRNA expression profiling analysis revealed pathways involved in the rapid growth of developing culms in Moso bamboo
Source: BMC Plant Biol. 2013 Aug 21;13:119. doi: 10.1186/1471-2229-13-119 (PMC3765735; doi:10.1186/1471-2229-13-119)

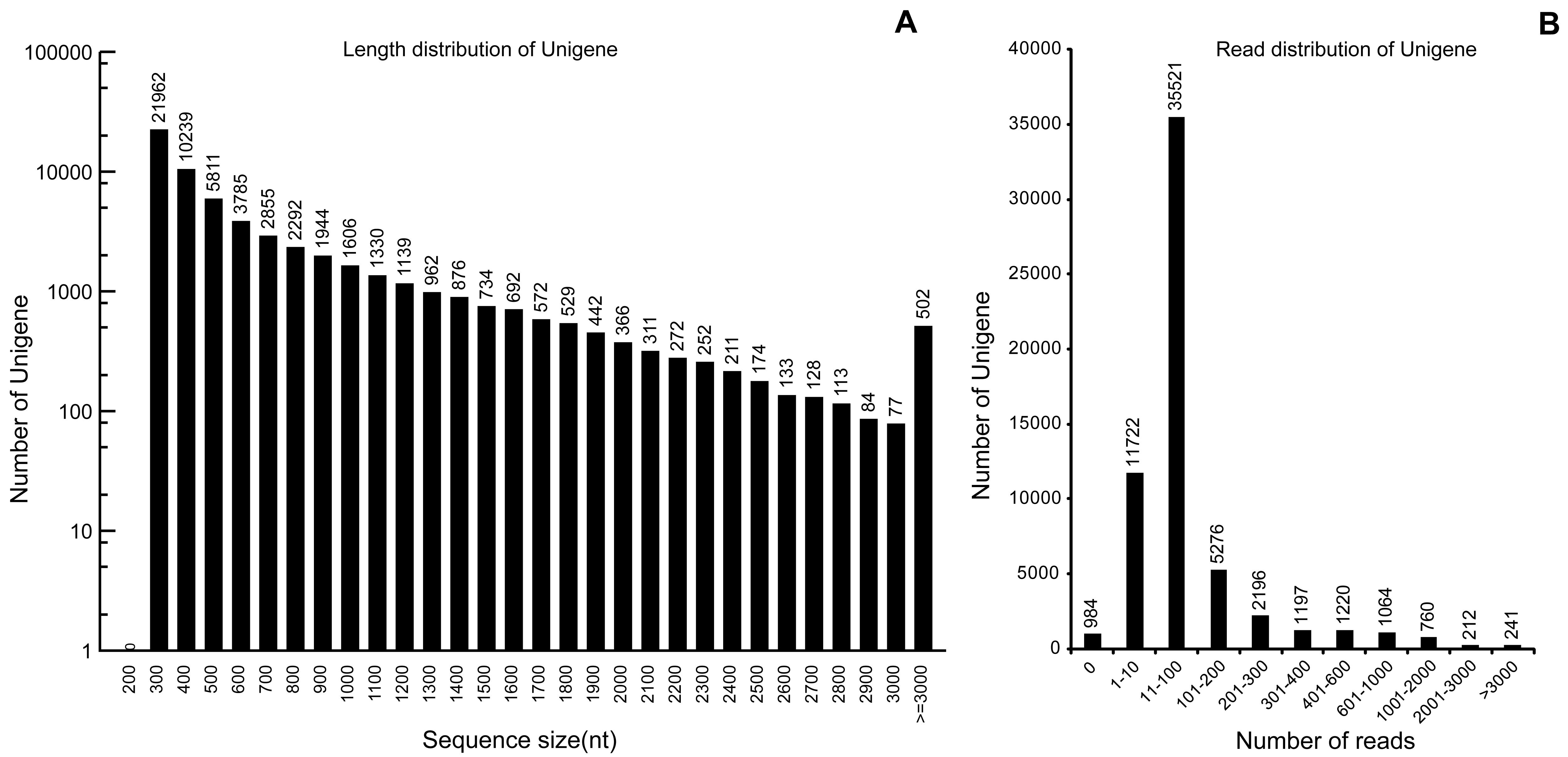

Supplement: Additional file 1: Figure S1 — Quality and coverage evaluation of assembled unigenes. Distribution of unique-mapped reads of the assembled unigenes. A: Length distribution of unigene. B: Read distribution of unigene. [file 1471-2229-13-119-S1.jpeg]

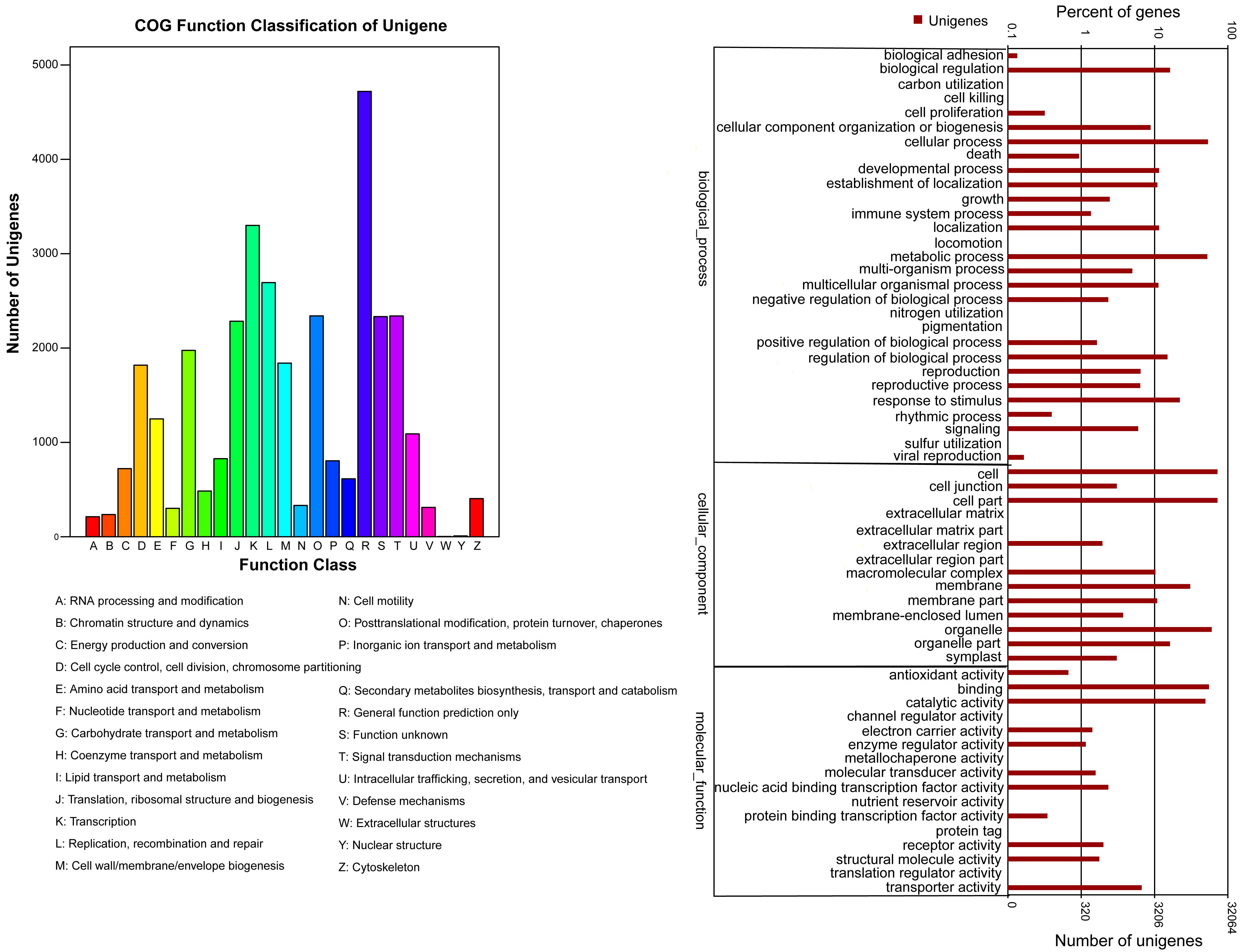

Supplement: Additional file 2: Figure S2 — Gene Ontology (right) and clusters of orthologous groups (COG) (left) classification of assembled unigenes. A total of 32,064 unigenes with BLAST matches to known proteins were assigned to three main categories: biological process, cellular component and molecular function. Out of 42,127 Nr hits, 13,957 sequences were assigned to 25 COG classifications. [file 1471-2229-13-119-S2.jpeg]

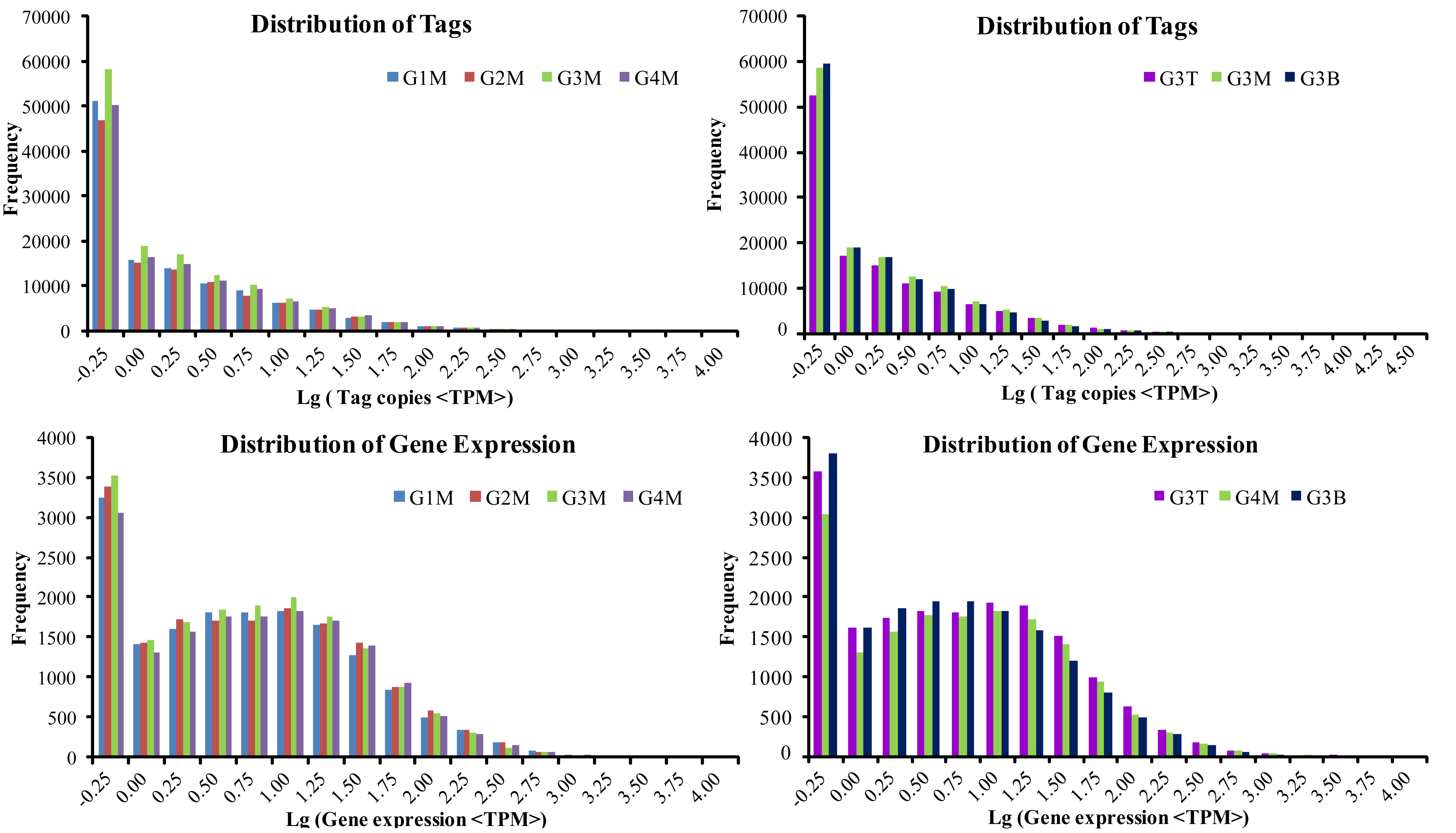

Supplement: Additional file 4: Figure S3 — Distribution of tags and gene expression among G1M-G4M, G3T and G3B groups. The distribution of tags matches that of gene expression among groups. Furthermore, an increase in tags or gene expression is accompanied by a decrease in the frequencies of tags or genes expression. [file 1471-2229-13-119-S4.jpeg]

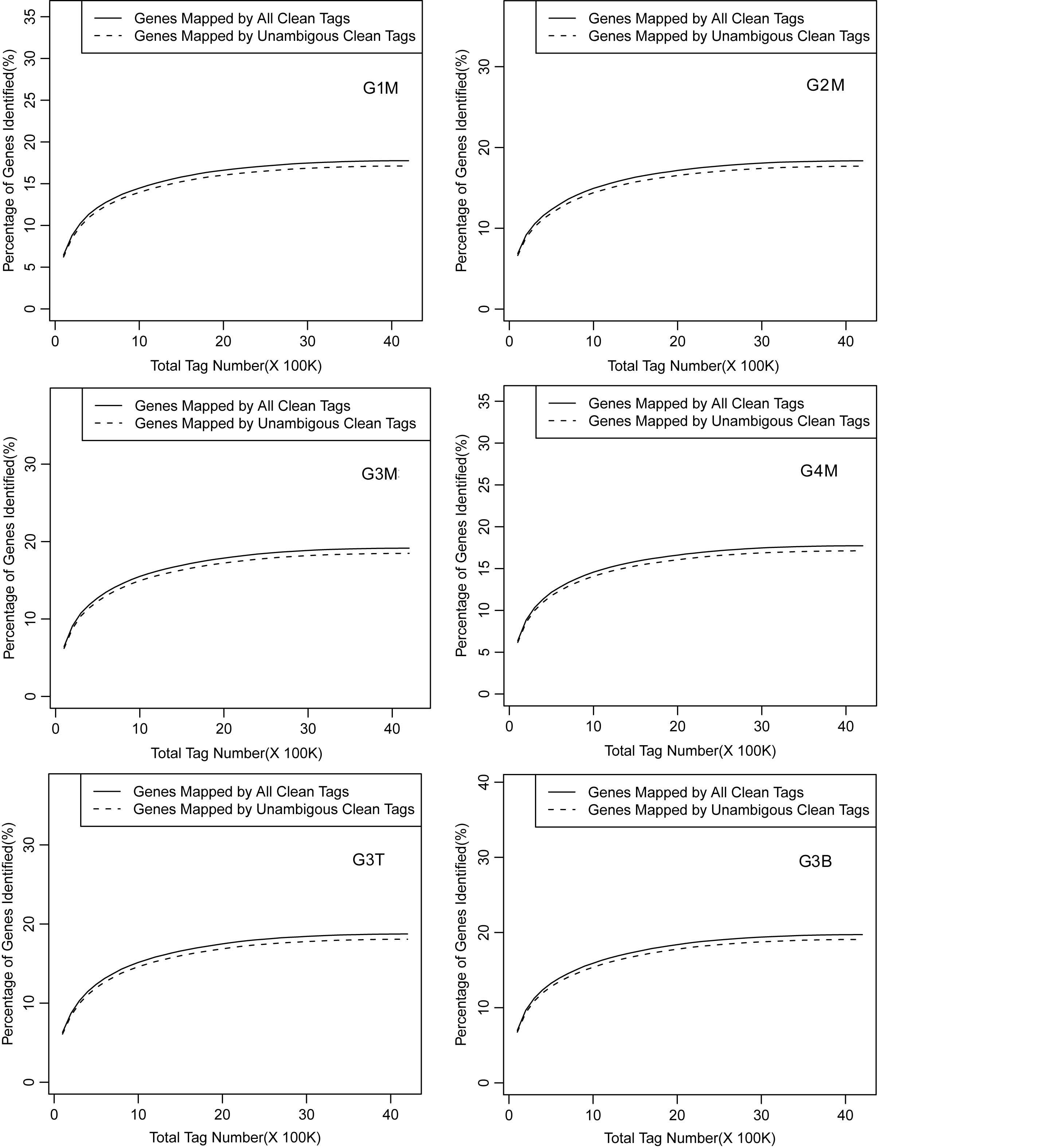

Supplement: Additional file 6: Figure S4 — Sequencing saturation evaluation of G1M-G4M, G3T and G3B libraries. [file 1471-2229-13-119-S6.jpeg]

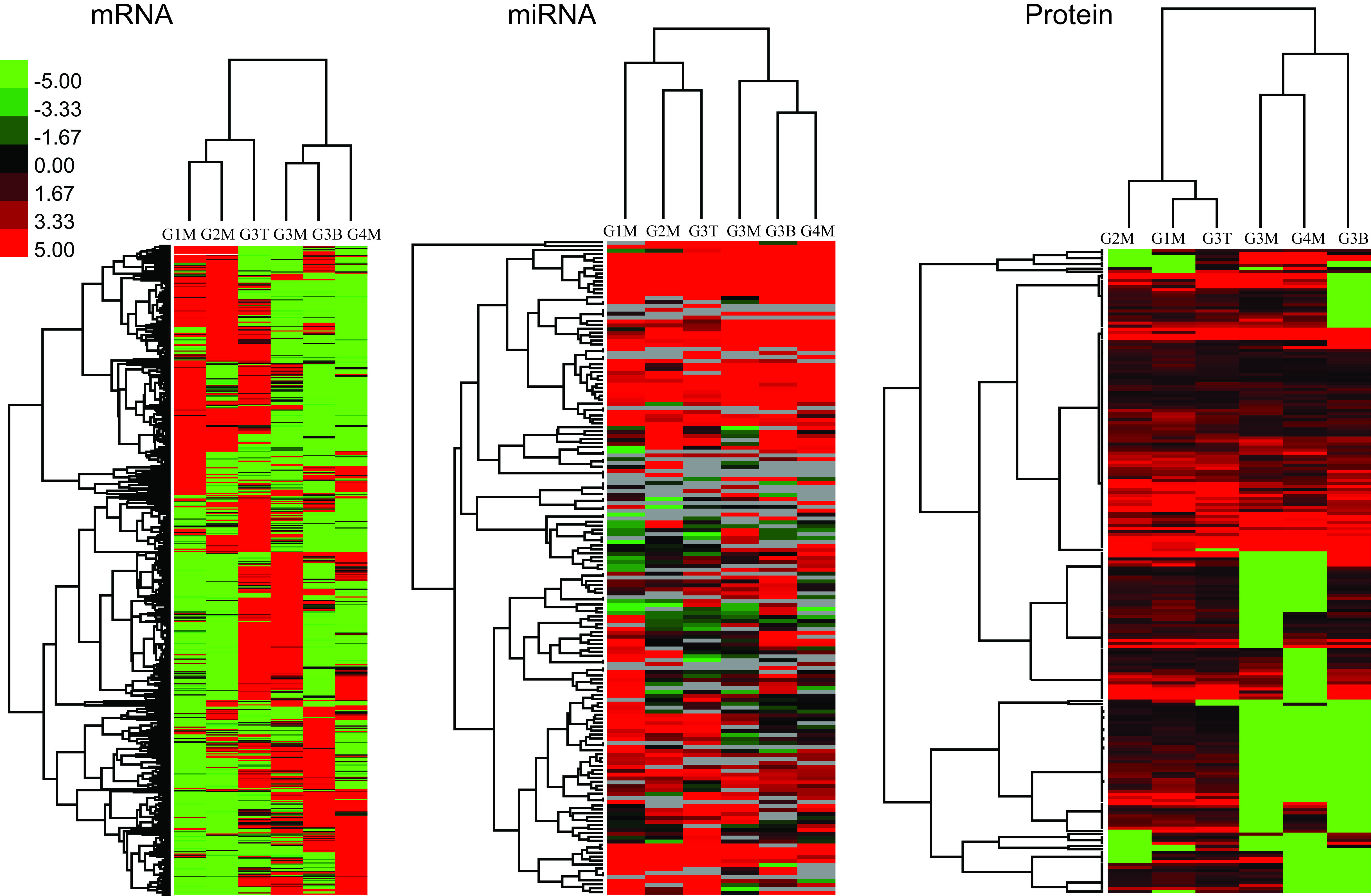

Supplement: Additional file 7: Figure S5 — Hierarchical clustering of differentially expressed genes, miRNAs and proteins. A total of 213 differentially expressed proteins (DEPs) were detected by two-dimensional gel electrophoresis (2-DE) and identified by matrix-assisted laser desorption/ionization time-of-flight/time-of-flight mass spectrometry (MALDI-TOF/TOF MS). G1M, G2M, G3M and G4M represent four developmental stages in turn. G3T, G3M and G3B represent top, middle and basal internode of the third developmental stage, respectively. The color ranges from green to red for the down-regulated and up-regulated genes, respectively. [file 1471-2229-13-119-S7.jpeg]
